# Revisiting the role of electron donors in lytic polysaccharide monooxygenase biochemistry

**DOI:** 10.1042/EBC20220164

**Published:** 2023-04-18

**Authors:** Glyn R. Hemsworth

**Affiliations:** Astbury Centre for Structural Molecular Biology and School of Molecular and Cellular Biology, Faculty of Biological Sciences, University of Leeds, Leeds LS2 9JT, U.K.

**Keywords:** electron donor, Lytic polysaccharide monooxygenase, oxidation-reduction

## Abstract

The plant cell wall is rich in carbohydrates and many fungi and bacteria have evolved to take advantage of this carbon source. These carbohydrates are largely locked away in polysaccharides and so these organisms deploy a range of enzymes that can liberate individual sugars from these challenging substrates. Glycoside hydrolases (GHs) are the enzymes that are largely responsible for bringing about this sugar release; however, 12 years ago, a family of enzymes known as lytic polysaccharide monooxygenases (LPMOs) were also shown to be of key importance in this process. LPMOs are copper-dependent oxidative enzymes that can introduce chain breaks within polysaccharide chains. Initial work demonstrated that they could activate O_2_ to attack the substrate through a reaction that most likely required multiple electrons to be delivered to the enzyme. More recently, it has emerged that LPMO kinetics are significantly improved if H_2_O_2_ is supplied to the enzyme as a cosubstrate instead of O_2_. Only a single electron is required to activate an LPMO and H_2_O_2_ cosubstrate and the enzyme has been shown to catalyse multiple turnovers following the initial one-electron reduction of the copper, which is not possible if O_2_ is used. This has led to further studies of the roles of the electron donor in LPMO biochemistry, and this review aims to highlight recent findings in this area and consider how ongoing research could impact our understanding of the interplay between redox processes in nature.

## Introduction

How fungi and bacteria are able to degrade cellulose and access the sugars contained within has intrigued biochemists for over 70 years [1]. Cellulose is a polymer of glucose in which individual sugar monomers are linked together by β-1,4-glycosidic linkages generating long polymeric chains that pack together into a highly crystalline and difficult to degrade structure. Research into the mechanisms by which this substrate can be degraded has not only been important to help understand carbon cycling but has also been industrially significant as we try to move away from our dependence on fossil fuels by enhancing the production of alternatives such as lignocellulosic biofuels (reviewed in [2,3]). We now have a very good understanding of the range of enzymes that some fungi and bacteria secrete in this process, which has aided the development of enzyme cocktails that can be deployed in the biorefinery (see [3–5] for reviews). The enzymes used have complementary activities that synergise with one another to bring about the depolymerisation of cellulose into individual glucose monomers. This enzymatic depolymerisation of cellulose was largely considered to be catalysed by glycoside hydrolases (GHs), collectively termed cellulases, which catalyse glycoside bond cleavage through the activation of water as a nucleophile [5]. About 12 years ago, however, a previously less well-understood enzyme class was discovered to play a key role in this process – lytic polysaccharide monooxygenases (LPMOs) [6–8].

LPMOs have been demonstrated to significantly improve the efficiency of cellulose (and other polysaccharide) degradation by GHs [6–11]. They catalyse the addition of a single atom of oxygen at either the C1 or C4 position of the sugar ring within polysaccharide chains, thereby destabilising the glycosidic linkage and leading to bond breakage [6,7,12–18]. By specifically acting within the more crystalline regions which can be difficult for GHs to access, LPMOs are thought to enhance GH activity by making the cellulose a more accessible substrate for these enzymes [6,7,12,14,15,19,20]. Given the dramatic enhancements in the efficiency of cellulose degradation as a result of LPMO action, there has been a considerable drive to understand the molecular mechanisms by which they function (see [21–24] for recent reviews).

**Figure 1:**
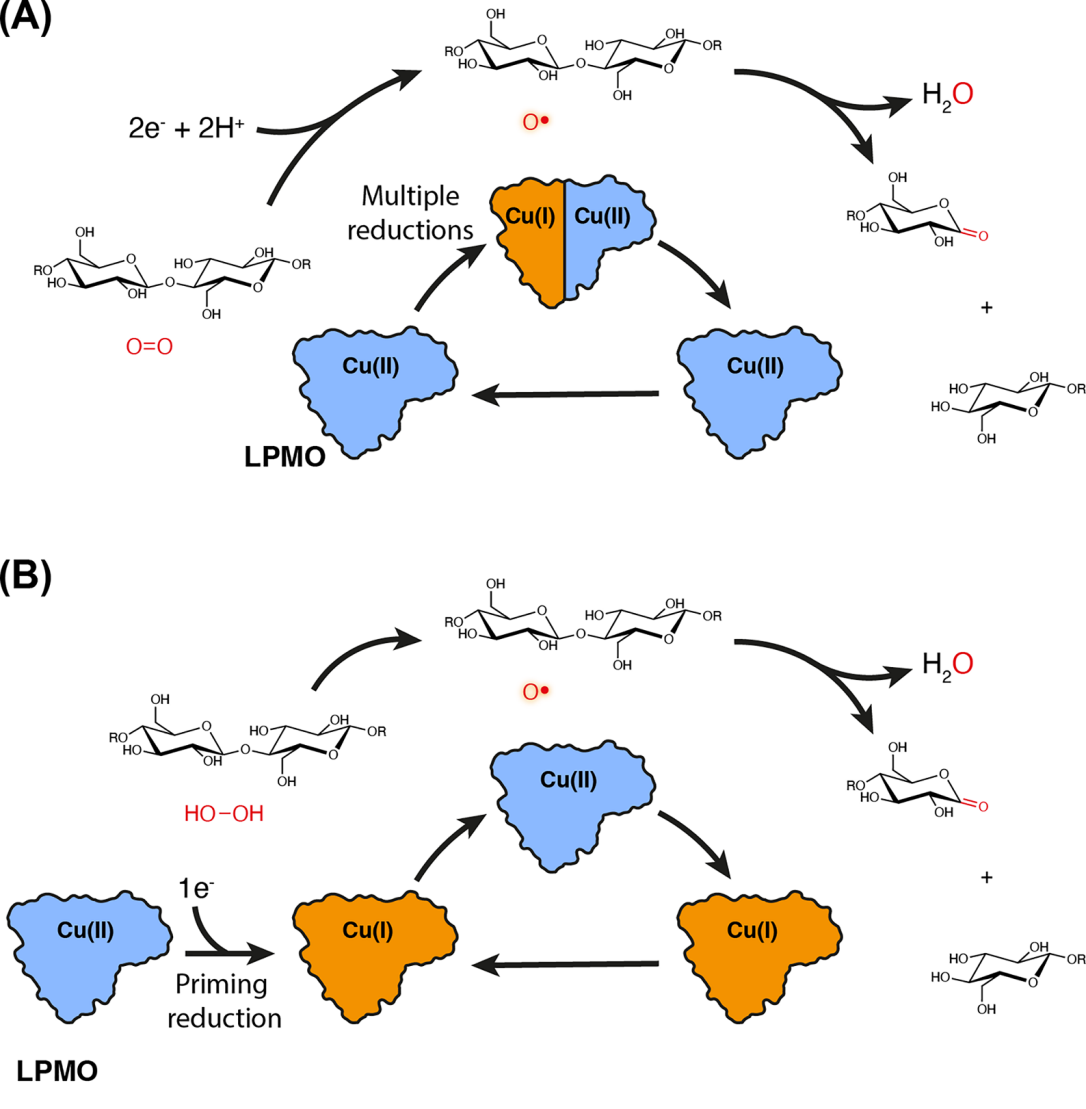
Comparison of the O_2_ and H_2_O_2_ dependent LPMO reactions. Schematic drawings illustrating the differences when (**A**) O_2_ is used as the LPMO cosubstrate as opposed to (**B**) H_2_O_2_. In both panels, reaction intermediates are shown along the outside with the redox state of the LPMO shown on the inside. For the LPMO to activate oxygen, it must undergo multiple reductions by continually receiving electrons from an electron donor. If H_2_O_2_ is used, an initial priming reduction is sufficient to catalyse the reaction and the enzyme can undergo multiple turnovers as it is returned to the Cu(I) state at the end of the reaction.

The LPMO active site contains a single copper ion that is bound by a motif known as the histidine brace [6,25–28]. This is formed by two histidine residues, one of which is the N-terminal residue, with the amino group providing one of the copper co-ordinating ligands and nitrogen atoms from the histidine sidechains providing the other two. Initial studies demonstrated that LPMOs used O_2_ as their cosubstrate and required a reducing agent to act as an electron donor to drive the reaction [6,7,29] (Figure 1A). Computational studies subsequently confirmed that multiple electrons were likely to be required to generate a sufficiently oxidising species to bring about hydrogen atom abstraction from either the C1 or C4 position of the sugar ring and allow hydroxylation of the substrate [30,31]. Copper-dependent enzymes typically require more than one copper cofactor to catalyse such multielectron reductions since copper can only accept a single electron into its outer shell. This left a conundrum for how LPMOs were catalysing this challenging chemistry given their active site architecture. More recent research has demonstrated that LPMO kinetics can be considerably improved by supplying the enzyme with H_2_O_2_ [13,32–34]. Not only are the kinetics more rapid but LPMOs can also catalyse multiple turnovers, following an initial reduction in the copper ion under such reaction conditions (Figure 1B). This, coupled with computational studies which demonstrate that peroxide is a more energetically favourable cosubstrate in the LPMO reaction [35–38], has led to suggestions that H_2_O_2_, and not O_2_, is the correct cosubstrate utilised by these enzymes. The fact that LPMOs can themselves generate H_2_O_2_ together with the complex nature of the reaction and the environments in which these enzymes are found makes demonstrating this a real challenge [13,34,35,38–42]. Whether peroxide is the ‘true’ LPMO cosubstrate or provides a ‘reaction shunt’ in which H_2_O_2_ is a key reactive intermediate in a normally O_2_-dependent reaction continues to be debated in the field.

Whichever cosubstrate is utilised by LPMOs, an electron source is required to reduce the active site copper and initiate the oxidative reaction catalysed by these enzymes [7,13]. A diversity of electron donors have been demonstrated as capable of driving the LPMO reaction, be it small-molecule reducing agents [7,10,16,17,43–49], other redox enzymes [29,41,50–53], or light-absorbing compounds from biotic or abiotic sources [54–57]. Some of these electron donors generate H_2_O_2_ themselves, which has now been implicated in several studies as responsible for what was previously considered the O_2_-dependent activity of LPMOs [33,43,58]. The aim of this review is to highlight recent findings in this area and to consider how these results impact the potential roles of other redox enzymes that have been implicated as electron donors in LPMO biochemistry. Understanding the interplay between enzymes, and how LPMOs are reduced in their native environment, could not only feed into the debate over the true nature of these enzymes but may also hold key insights into how LPMOs can be best harnessed industrially.

## Recent insights into the role of small-molecule electron donors

Early work on LPMOs demonstrated that a reducing agent was essential for catalysis [6,7]. Ascorbate is by far the most common reducing agent that is used in the laboratory for LPMO activity assays, but other small-molecule reducing agents like gallic acid, cysteine, lignin components, and a diverse array of other compounds have also been demonstrated as capable drivers of the LPMO reaction [7,10,16,17,44–49,59]. Indeed, before the realisation of the potential importance of peroxide in LPMO biochemistry, considerable variation in the levels of LPMO activity could be observed dependent upon the reducing agent used in the reaction [6,7,10,49]. It was reasonable to assume that these differences in activity related to the efficiency of the electron transfer to the LPMO active site, which could be influenced by the presence and identity of the substrate, amongst other factors. In addition, LPMOs were known to produce H_2_O_2_ in the absence of substrate, a property that could be used to help assay the enzymes and investigate their substrate specificities [42,60].

Recent research has revisited some of our assumptions relating to the role of the reductant in these enzyme assays with particular emphasis on the potential importance of H_2_O_2_ in the LPMO reaction. Many reducing agents will oxidise over time and can concomitantly produce H_2_O_2_ via the reduction of O_2_ [43,58]. Kuusk et al. performed a detailed kinetic analysis of LPMO activity on chitin nanowhiskers in which they examined ascorbate, gallic acid, and methylhydroquinone as electron donors in the presence of H_2_O_2_ [33]. Using a kinetic framework, they were able to demonstrate that the effectiveness of the reducing agent in driving the LPMO reaction was dependent upon its ability to reduce the copper active site and hence prime the enzyme for activity. This needed to be balanced against the inherent ability of the reducing agent itself to oxidise and potentially produce H_2_O_2_, with differing background levels of LPMO activity apparently being dependent upon the proclivity of the reducing agent to oxidise and produce H_2_O_2_ itself [33]. This was followed up by Stepnov et al. [43], who demonstrated that ascorbate-generated significant amounts of H_2_O_2_ in LPMO reactions independent of the enzyme’s activity. This was particularly prominent if free copper ions were also present in solution. Gallic acid, on the other hand, did not produce large amounts of H_2_O_2_ in the presence of copper ions [43]. It did produce some H_2_O_2_ in the absence of enzyme, and when incubated with the LPMO in the absence of substrate a linear increase in H_2_O_2_ production was observed. The authors reasoned that, at the enzyme concentrations used in LPMO assays, the amount of peroxide produced by the enzyme was likely insignificant compared with that generated by the oxidation of the gallic acid, thus the inherent oxidation of the reductant was producing H_2_O_2_ that would be used by the enzyme in its reaction. This rate of H_2_O_2_ production was therefore implicated as rate limiting in most LPMO reactions [43].

Another aspect of recent research in this area has further highlighted the large diversity of molecules that LPMOs can use as reductants and/or H_2_O_2_ sources in reactions with ‘real biomass.’ For instance, it has long been known that lignin components could be used to drive LPMO reactions [41,59]. Kuusk et al. also demonstrated that there may be molecules present within other biomasses (i.e. chitin) that could also impact LPMO kinetics [32]. The roles of such biomass compounds have recently been further considered in the context of light-driven reactions. Chlorophyllin was first shown as capable of driving LPMO reactions in the presence of a small amount of reducing agent in a light-dependent reaction [54]. Since then, other compounds including vanadium dioxide surfaces have also been demonstrated as capable of driving LPMOs in a light-dependent fashion [56]. It was initially considered that the inherent ability of these systems to generate electrons following light absorption was driving the LPMO reaction. Indeed, Mollers et al. demonstrated that reactive oxygen species (ROS)-scavenging enzymes had little effect on the light-driven LPMO reaction, implying that it was electron transfer from the photoactivatable pigment and not the generation of ROS that was driving the reaction [61]. Recent work, however, opposes this view as Bissaro et al. were able to show that H_2_O_2_ was generated in the absence of reducing agent as a result of light absorption by these compounds and that LPMO action could be powered under such conditions [55]. The subsequent activity of the LPMO was greatly enhanced by the presence of ascorbate feeding into a complex reaction in which the likely primary role of the light-absorbing pigment was in generating H_2_O_2_ and the initial reduction in the LPMO was triggered by the ascorbate [55]. Recent work has furthered this view by showing that phenolic compounds present within chitinaceous biomass also produce H_2_O_2_ in a light-dependent manner, which may also drive LPMO reactions under such conditions [57].

The emergent picture is therefore a complex one in which there is a balance between multiple processes in driving both the reduction of the LPMO active site and the generation of H_2_O_2_ to be harnessed by the LPMO. The remainder of the review will revisit research into other enzymes that have been implicated as capable of driving LPMO reactions and how the above findings may impact how we think about the interplay between such enzymes.

## Enzymes as electron donors to LPMOs in fungi

**Figure 2:**
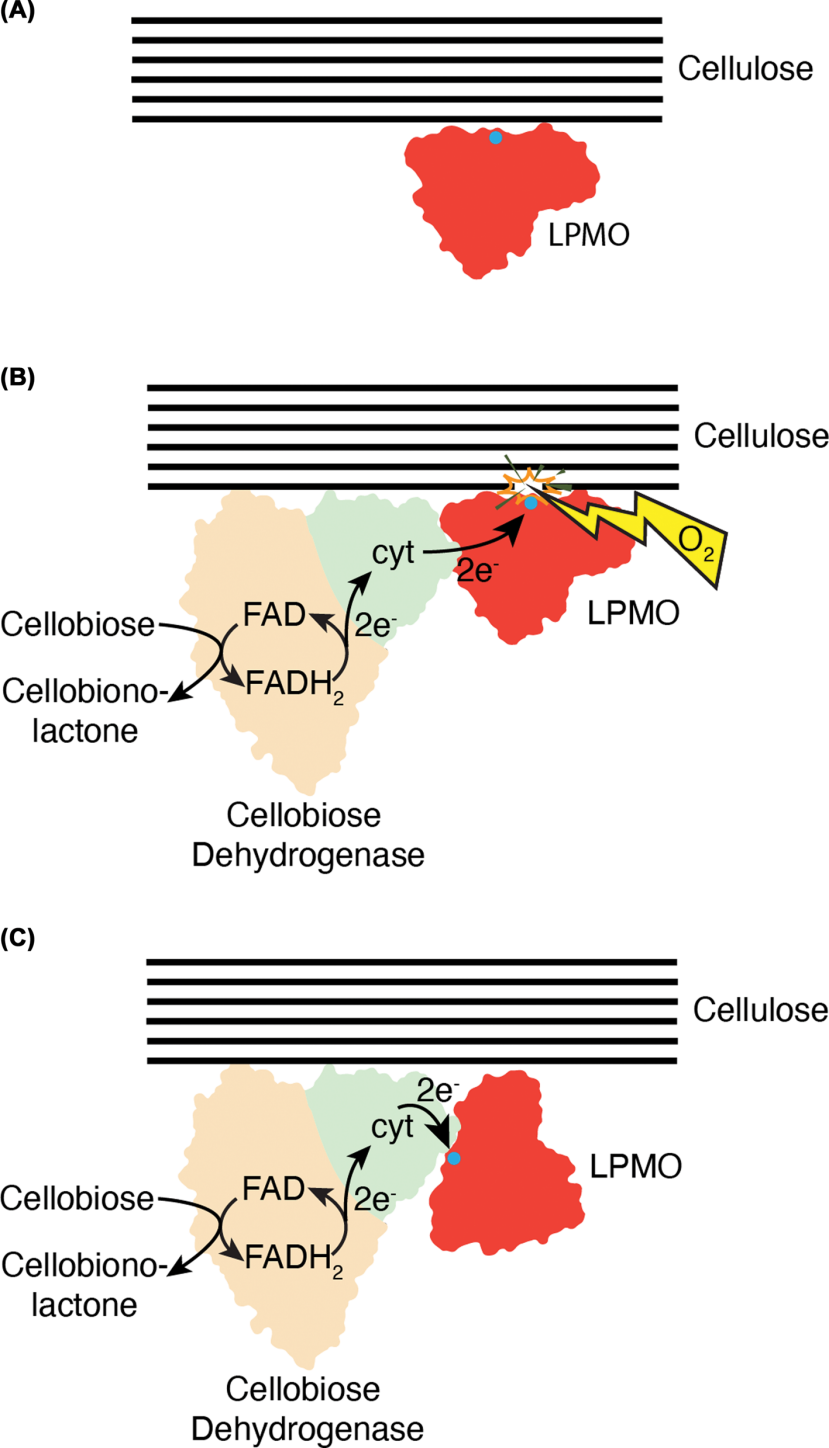
The relationship between the accessibility of the LPMO active site and how it can receive electrons from an enzyme partner. (**A**) The LPMO (red) when bound to crystalline cellulose (black lines) has its active site (blue circle) blocked. (**B**) By using an enzyme partner in the form of CDH (orange and green) as an electron source, it was considered that this would allow electron transfer at a position away from the active site, thereby allowing the reaction to proceed without the need for the LPMO to release the substrate. (**C**) Most data suggest that the LPMO is likely to contact the CDH cytochrome directly at the active site, which would necessitate several substrate binding and unbinding events for the O_2_-dependent reaction to proceed, so that sufficient electrons could be delivered to the enzyme.

Cellobiose dehydrogenase (CDH) is a member of the GMC oxidoreductase family of enzymes, these use FAD as a cofactor to oxidise an array of carbohydrate and other small-molecule substrates (see [62,63] for a reviews). CDH is unusual compared with other GMC oxidoreductases as it has a *b-*type cytochrome domain at its N-terminus, which has been implicated in shuttling electrons away to electron acceptors, allowing the FAD to be reoxidised to allow subsequent catalytic cycles [64,65]. Before the discovery of LPMOs, CDH was known to play an important role in cellulose utilisation but was largely considered to be required to generate H_2_O_2_ to be used in a nonenzymatic Fenton reaction used by some fungi to drive cellulose deconstruction [66,67]. Following the discovery of LPMOs, CDH was also demonstrated to be capable of driving the LPMO reaction in place of small-molecule reducing agents [29,50]. Indeed, direct electron transfer between CDH and an LPMO was shown to occur experimentally and was mediated by the CDH *b*-type cytochrome domain [51]. Having an enzyme partner to drive the LPMO monooxygenase reaction, which requires two-electrons, was an attractive prospect given the conundrum over how multiple electrons could be delivered to the LPMO active site when it was likely blocked by its substrate (Figure 2A,B). This led to suggestions of potential electron transfer pathways that may be present within the core of LPMOs, which would allow distal interactions with an electron-donating protein partner [10,68]. Structures of CDH in different conformations with the cytochrome either docked against the dehydrogenase domain or extending away to interact with an electron acceptor suggested that the dynamics of CDH may play into such an interaction [64,69]. However, attempts to detect protein–protein interactions between these proteins, either experimentally or via computational docking, favour a model in which the cytochrome domain directly contacts the LPMO copper active site, thereby necessitating the enzyme being devoid of its polysaccharide substrate in order to receive electrons from CDH (Figure 2C) [64,70,71].

The mode of interaction between these enzymes could feed into the peroxygenase/monooxygenase debate for LPMOs. Given its ability to both generate H_2_O_2_ and transfer electrons via its cytochrome domain [29,50,66,67], CDH would appear to be the perfect enzyme to power LPMOs in fungi. Indeed, it was proposed following the discovery of the interaction between LPMOs and CDH that the true role of CDH may be to work in harness with LPMOs in biomass breakdown [29,51]. Recently, stopped flow kinetics have been used by Hedison and co-workers to gain kinetic insight into the electron transfer from a reduced CDH cytochrome domain to the LPMO under H_2_O_2_-containing conditions [72]. As was the case with small-molecule reducing agents, the authors found that a single-electron-donation event from the cytochrome was sufficient to catalyse multiple turnovers of the LPMO in the presence of peroxide. If H_2_O_2_ is thus the cosubstrate, this may alleviate the need for a through-protein electron-transfer pathway in the LPMO to allow its activity whilst bound to substrate. An alternative function for the internal electron transfer routes within LPMOs has recently been suggested [73]. During studies on a redox species generated on an LPMO characterised by its purple colour, it was suggested that these pathways may be important to allow potentially damaging ‘holes’ to be shuttled away from the active site if there is not a productive interaction with the substrate, thereby protecting the active site [73]. This ‘hole-hopping’ hypothesis was furthered by Jones et al., who also demonstrated the existence of radicals on two amino acids in another LPMO [74]. Such noncatalytic pathways may, thus, represent a general feature of these enzymes that provides a mechanism towards ensuring their longevity and maximising their catalytic potential.

Not all LPMO-producing fungi produce a CDH, which has generated interest in identifying alternative electron sources in such fungi. This has led to demonstrations of other GMC oxidoreductases that lack cytochrome domains, e.g. glucose dehydrogenase, being capable of driving LPMO reactions through small-molecule mediators [41,52]. A new class of PQQ-dependent dehydrogenase has also been defined [75]. These often have an appended *b*-type cytochrome domain with sequence similarity to the domain present in CDH [75]. A protein in this class has been demonstrated as a pyranose dehydrogenase, which was capable of powering an LPMO to catalyse its reaction before the realisation that LPMOs might be peroxide dependent [53]. It is not clear whether these enzymes also generate H_2_O_2_, which could be harnessed by the LPMO but it is known that the reduced form of PQQ (PQQH_2_) can be oxidised back to its native state in air thus producing H_2_O_2_ [76]. It is, therefore, likely that such enzymes may act in place of CDH in some fungi. In a further recent advance, several fungal FAD-dependent oligosaccharide oxidases have also been shown to support LPMO activity [77]. These enzymes were shown to be active on a range of oligosaccharides, and not just those derived from cellulose or chitin, diversifying the pool of substrates that could be used to drive LPMO reactions. These enzymes can also generate H_2_O_2_ and appear to directly transfer electrons to LPMOs in a nonspecific manner as demonstrated by EPR [77]. Interestingly, these enzymes lack a cytochrome domain that has been implicated in electron transfer in the other redox enzymes discussed earlier.

The emerging picture is one in which there is a diversity of fungal redox enzymes that can act in harness with LPMOs outside of the cell. How these enzymes interact, and work together in their native environment is currently unclear, however. Fungal growth is complex, thus, unravelling the interplay between enzymes during this process is a major challenge. Nonetheless, if such insight could be gained, then this could significantly help in delineating the true nature of LPMOs and it could inform on how LPMOs could be best used in industry to ensure that enzyme lifetimes are maximised.

## Protein-based electron donors from bacteria

Fungi, and the enzymes that they produce, are most heavily studied in the context of polysaccharide utilisation and LPMO biochemistry. However, bacteria also make use of LPMOs, but they do not encode an enzyme equivalent to CDH within their genomes and so there have been some attempts to identify potential protein-based electron-transfer proteins from such microbes. Cbp2D and Cbp2E from *Cellvibrio japonicus* are the main proteins that have been considered in this context thus far [78]. Deletion of the genes coding for these proteins led to an impairment of the bacterium’s ability to grow on cellulose filter paper, though the same phenotype was not observed when the individual LPMO genes were deleted. The proteins encoded for by these genes have also been detected in the secretome during growth on chitin [79], demonstrating a likely role in polysaccharide utilisation beyond cellulose. Sequence analysis of the predicted amino acid sequences for these proteins show that they both contain domains that are related to the YceI class of lipocalin like proteins [78,79], or X158 domains as they can be referred to in the CAZyme-specific literature [80]. A single X158 has been characterised to date but it, as well as many other distantly-related domains, have been purified with the well-known redox molecule ubiquinone-8 bound, as revealed by their crystal structures [80]. In addition, Cbp2D is also predicted to contain cytochrome-like domains at the C-terminus of the protein, which would suggest a likely electron-transfer function, hence, an interest in them as potential LPMO electron donors [78,81]. In our own recent work, we were able to isolate one of the cytochrome-like domains (*Cj*X183D) from Cbp2D and showed that it was likely an electron transferring, as opposed to catalytic, *c*-type cytochrome [81]. Its reduced state was able to drive LPMO catalysis in the presence of O_2_ but activity in the presence of H_2_O_2_ was not considered at the time. Rather H_2_O_2_ production by the LPMO was used as a proxy to evaluate the ability of the domain to donate electrons to the enzyme, which revealed interesting differences between the apparent rates of electron transfer between the X183 and the *C. japonicus* LPMO when compared with an LPMO from another bacterial species [81]. This was considered as a possible indication of a specific interaction having evolved between the proteins though this requires further investigation. Whether such interactions exist or not, the apparent rates appeared to be too slow to prove an underlying biological function in electron donation to LPMOs. However, the results did highlight the potential utility for protein partners to be used either in industry or in biology as LPMO activators that cause lowered protein damage relative to synthetic chemical electron donors.

Whether so-called ‘X-domain’ and related proteins are involved in LPMO biochemistry, or if they have some other biological function in bacteria is still an open question. Genes that code for such proteins are not found in all LPMO-producing bacteria, and if H_2_O_2_ is the true cosubstrate for LPMOs, then it would appear unlikely that these complex proteins would be present purely to donate electrons to the LPMO. There is therefore much to still be discovered as to the underlying redox biochemistry that takes place outside of the bacterial cell during growth on complex polysaccharides.

## Conclusions

The question as to whether LPMOs are truly monooxygenases or peroxidases has revealed new insights into our assumptions over the role of the electron donor in driving LPMO catalysis. Recent findings using small-molecule reducing agents and light-driven reactions suggest that the proclivity for the reducing agent to generate H_2_O_2_ in laboratory conditions may be equally as important as its ability to reduce the active site copper on the enzyme to drive LPMO catalysis. Away from laboratory conditions, it is harder to know what the consequences of these findings are for how these enzymes are harnessed in nature. In fungi, CDH may be the perfect protein partner for powering LPMOs given its ability to both produce H_2_O_2_ and transfer electrons for reducing the copper active site. But thus far, there is not a clear electron-donor protein to bacterial LPMOs, nor is it known whether one is really required given the diversity of sources of H_2_O_2_ and electrons that can be encountered in nature. Both the LPMO reaction and the environment in which these enzymes function is a complex one. What is clear is that LPMOs are able to accept electrons from a diversity of compounds and proteins to allow their active site copper to be reduced and to drive the reaction. Whether H_2_O_2_ is the true cosubstrate or a reactive intermediate, the provision of which offers a shunt to the natural O_2_-dependent reaction, is challenging to dissect. Gaining a deeper understanding of how LPMOs are used during fungal and/or bacterial growth could considerably deepen our understanding of the redox processes that drive polysaccharide deconstruction in these organisms and feed into this debate more deeply. Furthermore, LPMOs are now a key component of industrial enzyme cocktails that are used in the biorefinery. By gaining a deeper understanding of the reductive processes that drive LPMO action, this can also inform on the best practice for harnessing these enzymes in industry.

## Summary

- LPMOs are important redox enzymes in biomass breakdown and there is ongoing debate as to their mechanism of action.
- The role of the electron donor is being reconsidered considering recent findings that H_2_O_2_ can significantly enhance the rate and number of turnovers LPMOs can undergo.
- The emerging picture is one in which the electron donor used by the LPMO may both generate H_2_O_2_ as the enzyme’s cosubstrate and reduce the LPMO active site, allowing it to perform its reaction.
- A plethora of electron donors have been demonstrated to drive the LPMO reaction, more work needs to be done to clarify the mode of interaction between such molecules, which may help clarify whether LPMOs are peroxygenases or monooxygenases.
- It is clear that LPMOs function in a complex redox environment in nature. To truly draw a line under the peroxygenase/monooxygenase debate, it will be necessary to gain deeper insight into how LPMOs function in their native environments.

## Abbreviations

CDH: cellobiose dehydrogenase
EPR: electron paramagnetic resonance
FAD: flavin adenine dinucleotide
GH: glycoside hydrolase
GMC: glucose-methanol-choline
LPMO: lytic polysaccharide monooxygenase
PQQ: pyrroloquinoline quinone
ROS: reactive oxygen species

## References

[1] Bischof R.H., Ramoni J. and Seiboth B. (2016) Cellulases and beyond: the first 70 years of the enzyme producer *Trichoderma reesei*. Microb Cell Fact 15, 106 10.1186/s12934-016-0507-6 27287427 PMC4902900

[2] Himmel M.E., Ding S.Y., Johnson D.K., Adney W.S., Nimlos M.R., Brady J.W. et al. (2007) Biomass recalcitrance: engineering plants and enzymes for biofuels production. Science 315, 804–807 10.1126/science.1137016 17289988

[3] Harris P.V., Xu F., Kreel N.E., Kang C. and Fukuyama S. (2014) New enzyme insights drive advances in commercial ethanol production. Curr. Opin. Chem. Biol. 19, 162–170 10.1016/j.cbpa.2014.02.015 24681544

[4] Horn S.J., Vaaje-Kolstad G., Westereng B. and Eijsink V.G. (2012) Novel enzymes for the degradation of cellulose. Biotechnol. Biofuels 5, 45 10.1186/1754-6834-5-45 22747961 PMC3492096

[5] Payne C.M., Knott B.C., Mayes H.B., Hansson H., Himmel M.E., Sandgren M. et al. (2015) Fungal cellulases. Chem. Rev. 115, 1308–1448 10.1021/cr500351c 25629559

[6] Quinlan R.J., Sweeney M.D., Lo Leggio L., Otten H., Poulsen J.C., Johansen K.S. et al. (2011) Insights into the oxidative degradation of cellulose by a copper metalloenzyme that exploits biomass components. Proc. Natl. Acad. Sci. U.S.A. 108, 15079–15084 10.1073/pnas.1105776108 21876164 PMC3174640

[7] Vaaje-Kolstad G., Westereng B., Horn S.J., Liu Z., Zhai H., Sorlie M. et al. (2010) An oxidative enzyme boosting the enzymatic conversion of recalcitrant polysaccharides. Science 330, 219–222 10.1126/science.1192231 20929773

[8] Harris P.V., Welner D., McFarland K.C., Re E., Navarro Poulsen J.C., Brown K. et al. (2010) Stimulation of lignocellulosic biomass hydrolysis by proteins of glycoside hydrolase family 61: structure and function of a large, enigmatic family. Biochemistry 49, 3305–3316 10.1021/bi100009p 20230050

[9] Cannella D., Hsieh C.W., Felby C. and Jorgensen H. (2012) Production and effect of aldonic acids during enzymatic hydrolysis of lignocellulose at high dry matter content. Biotechnol. Biofuels 5, 26 10.1186/1754-6834-5-26 22546481 PMC3458932

[10] Lo Leggio L., Simmons T.J., Poulsen J.C., Frandsen K.E., Hemsworth G.R., Stringer M.A. et al. (2015) Structure and boosting activity of a starch-degrading lytic polysaccharide monooxygenase. Nat. Commun. 6, 5961 10.1038/ncomms6961 25608804 PMC4338556

[11] Sabbadin F., Hemsworth G.R., Ciano L., Henrissat B., Dupree P., Tryfona T. et al. (2018) An ancient family of lytic polysaccharide monooxygenases with roles in arthropod development and biomass digestion. Nat. Commun. 9, 756 10.1038/s41467-018-03142-x 29472725 PMC5823890

[12] Bey M., Zhou S., Poidevin L., Henrissat B., Coutinho P.M., Berrin J.G. et al. (2013) Cello-oligosaccharide oxidation reveals differences between two lytic polysaccharide monooxygenases (family GH61) from *Podospora anserina*. Appl. Environ. Microbiol. 79, 488–496 10.1128/AEM.02942-12 23124232 PMC3553762

[13] Bissaro B., Rohr A.K., Muller G., Chylenski P., Skaugen M., Forsberg Z. et al. (2017) Oxidative cleavage of polysaccharides by monocopper enzymes depends on H_2_O_2_. Nat. Chem. Biol. 13, 1123–1128 10.1038/nchembio.2470 28846668

[14] Forsberg Z., Vaaje-Kolstad G., Westereng B., Bunaes A.C., Stenstrom Y., MacKenzie A. et al. (2011) Cleavage of cellulose by a CBM33 protein. Protein Sci. 20, 1479–1483 10.1002/pro.689 21748815 PMC3190143

[15] Westereng B., Ishida T., Vaaje-Kolstad G., Wu M., Eijsink V.G., Igarashi K. et al. (2011) The putative endoglucanase PcGH61D from *Phanerochaete chrysosporium* is a metal-dependent oxidative enzyme that cleaves cellulose. PloS ONE 6, e27807 10.1371/journal.pone.0027807 22132148 PMC3223205

[16] Beeson W.T., Phillips C.M., Cate J.H. and Marletta M.A. (2012) Oxidative cleavage of cellulose by fungal copper-dependent polysaccharide monooxygenases. J. Am. Chem. Soc. 134, 890–892 10.1021/ja210657t 22188218

[17] Isaksen T., Westereng B., Aachmann F.L., Agger J.W., Kracher D., Kittl R. et al. (2014) A C4-oxidizing lytic polysaccharide monooxygenase cleaving both cellulose and cello-oligosaccharides. J. Biol. Chem. 289, 2632–2642 10.1074/jbc.M113.530196 24324265 PMC3908397

[18] Sun P., Laurent C., Boerkamp V.J.P., van Erven G., Ludwig R., van Berkel W.J.H. et al. (2022) Regioselective C4 and C6 double oxidation of cellulose by lytic polysaccharide monooxygenases. Chem. Sus. Chem. 15, e202102203 10.1002/cssc.202102203 34859958 PMC9299857

[19] Eibinger M., Ganner T., Bubner P., Rosker S., Kracher D., Haltrich D. et al. (2014) Cellulose surface degradation by a lytic polysaccharide monooxygenase and its effect on cellulase hydrolytic efficiency. J. Biol. Chem. 289, 35929–35938 10.1074/jbc.M114.602227 25361767 PMC4276861

[20] Arslan E., Schulz H., Zufferey R., Kunzler P. and Thony-Meyer L. (1998) Overproduction of the *Bradyrhizobium japonicum c*-type cytochrome subunits of the cbb3 oxidase in *Escherichia coli*. Biochem. Biophys. Res. Commun. 251, 744–747 10.1006/bbrc.1998.9549 9790980

[21] Ciano L., Davies G.J., Tolman W.B. and Walton P.H. (2018) Bracing copper for the catalytic oxidation of C–H bonds. Nat. Catalysis 1, 571–577 10.1038/s41929-018-0110-9

[22] Forsberg Z., Sorlie M., Petrovic D., Courtade G., Aachmann F.L., Vaaje-Kolstad G. et al. (2019) Polysaccharide degradation by lytic polysaccharide monooxygenases. Curr. Opin. Struct. Biol. 59, 54–64 10.1016/j.sbi.2019.02.015 30947104

[23] Walton P.H. and Davies G.J. (2016) On the catalytic mechanisms of lytic polysaccharide monooxygenases. Curr. Opin. Chem. Biol. 31, 195–207 10.1016/j.cbpa.2016.04.001 27094791

[24] Ipsen J.O., Hallas-Moller M., Brander S., Lo Leggio L. and Johansen K.S. (2021) Lytic polysaccharide monooxygenases and other histidine-brace copper proteins: structure, oxygen activation and biotechnological applications. Biochem. Soc. Trans. 49, 531–540 10.1042/BST20201031 33449071 PMC7924993

[25] Gudmundsson M., Kim S., Wu M., Ishida T., Momeni M.H., Vaaje-Kolstad G. et al. (2014) Structural and electronic snapshots during the transition from a Cu(II) to Cu(I) metal center of a lytic polysaccharide monooxygenase by X-ray photoreduction. J. Biol. Chem. 289, 18782–18792 10.1074/jbc.M114.563494 24828494 PMC4081921

[26] Hemsworth G.R., Davies G.J. and Walton P.H. (2013) Recent insights into copper-containing lytic polysaccharide mono-oxygenases. Curr. Opin. Struct. Biol. 23, 660–668 10.1016/j.sbi.2013.05.006 23769965

[27] Hemsworth G.R., Taylor E.J., Kim R.Q., Gregory R.C., Lewis S.J., Turkenburg J.P. et al. (2013) The copper active site of CBM33 polysaccharide oxygenases. J. Am. Chem. Soc. 135, 6069–6077 10.1021/ja402106e 23540833 PMC3636778

[28] Vaaje-Kolstad G., Forsberg Z., Loose J.S., Bissaro B. and Eijsink V.G. (2017) Structural diversity of lytic polysaccharide monooxygenases. Curr. Opin. Struct. Biol. 44, 67–76 10.1016/j.sbi.2016.12.012 28086105

[29] Phillips C.M., Beeson W.T., Cate J.H. and Marletta M.A. (2011) Cellobiose dehydrogenase and a copper-dependent polysaccharide monooxygenase potentiate cellulose degradation by *Neurospora crassa*. ACS Chem. Biol. 6, 1399–1406 10.1021/cb200351y 22004347

[30] Kim S., Stahlberg J., Sandgren M., Paton R.S. and Beckham G.T. (2014) Quantum mechanical calculations suggest that lytic polysaccharide monooxygenases use a copper-oxyl, oxygen-rebound mechanism. Proc. Natl. Acad. Sci. U.S.A. 111, 149–154 10.1073/pnas.1316609111 24344312 PMC3890868

[31] Kjaergaard C.H., Qayyum M.F., Wong S.D., Xu F., Hemsworth G.R., Walton D.J. et al. (2014) Spectroscopic and computational insight into the activation of O_2_ by the mononuclear Cu center in olysaccharide monooxygenases. Proc. Natl. Acad. Sci. U.S.A. 111, 8797–8802 10.1073/pnas.1408115111 24889637 PMC4066490

[32] Kuusk S., Bissaro B., Kuusk P., Forsberg Z., Eijsink V.G.H., Sorlie M. et al. (2018) Kinetics of H_2_O_2_-driven degradation of chitin by a bacterial lytic polysaccharide monooxygenase. J. Biol. Chem. 293, 523–531 10.1074/jbc.M117.817593 29138240 PMC5767858

[33] Kuusk S., Kont R., Kuusk P., Heering A., Sorlie M., Bissaro B. et al. (2019) Kinetic insights into the role of the reductant in H_2_O_2_-driven degradation of chitin by a bacterial lytic polysaccharide monooxygenase. J. Biol. Chem. 294, 1516–1528 10.1074/jbc.RA118.006196 30514757 PMC6364757

[34] Müller G., Chylenski P., Bissaro B., Eijsink V.G.H. and Horn S.J. (2018) The impact of hydrogen peroxide supply on LPMO activity and overall saccharification efficiency of a commercial cellulase cocktail. Biotechnol. Biofuels 11, 209 10.1186/s13068-018-1199-4 30061931 PMC6058378

[35] Bissaro B., Streit B., Isaksen I., Eijsink V.G.H., Beckham G.T., DuBois J.L. et al. (2020) Molecular mechanism of the chitinolytic peroxygenase reaction. Proc. Natl. Acad. Sci. U.S.A. 117, 1504–1513 10.1073/pnas.1904889117 31907317 PMC6983374

[36] Wang B., Johnston E.M., Li P., Shaik S., Davies G.J., Walton P.H. et al. (2018) QM/MM studies into the H_2_O_2_-dependent activity of lytic polysaccharide monooxygenases: evidence for the formation of a caged hydroxyl radical intermediate. ACS Catalysis 8, 1346–1351 10.1021/acscatal.7b03888

[37] Wang B., Walton P.H. and Rovira C. (2019) Molecular mechanisms of oxygen activation and hydrogen peroxide formation in lytic polysaccharide monooxygenases. ACS Catalysis 9, 4958–4969 10.1021/acscatal.9b00778 32051771 PMC7007194

[38] Caldararu O., Oksanen E., Ryde U. and Hedegard E.D. (2019) Mechanism of hydrogen peroxide formation by lytic polysaccharide monooxygenase. Chem. Sci. 10, 576–586 10.1039/C8SC03980A 30746099 PMC6334667

[39] Hangasky J.A., Iavarone A.T. and Marletta M.A. (2018) Reactivity of O_2_ versus H_2_O_2_ with polysaccharide monooxygenases. Proc. Natl. Acad. Sci. U.S.A. 115, 4915–4920 10.1073/pnas.1801153115 29686097 PMC5949000

[40] Kont R., Bissaro B., Eijsink V.G.H. and Valjamae P. (2020) Kinetic insights into the peroxygenase activity of cellulose-active lytic polysaccharide monooxygenases (LPMOs). Nat. Commun. 11, 5786 10.1038/s41467-020-19561-8 33188177 PMC7666214

[41] Kracher D., Scheiblbrandner S., Felice A.K., Breslmayr E., Preims M., Ludwicka K. et al. (2016) Extracellular electron transfer systems fuel cellulose oxidative degradation. Science 352, 1098–1101 10.1126/science.aaf3165 27127235

[42] Kittl R., Kracher D., Burgstaller D., Haltrich D. and Ludwig R. (2012) Production of four *Neurospora crassa* lytic polysaccharide monooxygenases in *Pichia pastoris* monitored by a fluorimetric assay. Biotechnol. Biofuels 5, 79 10.1186/1754-6834-5-79 23102010 PMC3500269

[43] Stepnov A.A., Forsberg Z., Sorlie M., Nguyen G.S., Wentzel A., Rohr A.K. et al. (2021) Unraveling the roles of the reductant and free copper ions in LPMO kinetics. Biotechnol. Biofuels 14, 28 10.1186/s13068-021-01879-0 33478537 PMC7818938

[44] Sabbadin F., Pesante G., Elias L., Besser K., Li Y., Steele-King C. et al. (2018) Uncovering the molecular mechanisms of lignocellulose digestion in shipworms. Biotechnol. Biofuels 11, 59 10.1186/s13068-018-1058-3 29527236 PMC5840672

[45] Couturier M., Ladeveze S., Sulzenbacher G., Ciano L., Fanuel M., Moreau C. et al. (2018) Lytic xylan oxidases from wood-decay fungi unlock biomass degradation. Nat. Chem. Biol. 14, 306–310 10.1038/nchembio.2558 29377002

[46] Filiatrault-Chastel C., Navarro D., Haon M., Grisel S., Herpoel-Gimbert I., Chevret D. et al. (2019) AA16, a new lytic polysaccharide monooxygenase family identified in fungal secretomes. Biotechnol. Biofuels 12, 55 10.1186/s13068-019-1394-y 30923563 PMC6420742

[47] Hemsworth G.R., Henrissat B., Davies G.J. and Walton P.H. (2014) Discovery and characterization of a new family of lytic polysaccharide monooxygenases. Nat. Chem. Biol. 10, 122–126 10.1038/nchembio.1417 24362702 PMC4274766

[48] Vu V.V., Beeson W.T., Span E.A., Farquhar E.R. and Marletta M.A. (2014) A family of starch-active polysaccharide monooxygenases. Proc. Natl. Acad. Sci. U.S.A. 111, 13822–13827 10.1073/pnas.1408090111 25201969 PMC4183312

[49] Simmons T.J., Frandsen K.E.H., Ciano L., Tryfona T., Lenfant N., Poulsen J.C. et al. (2017) Structural and electronic determinants of lytic polysaccharide monooxygenase reactivity on polysaccharide substrates. Nat. Commun. 8, 1064 10.1038/s41467-017-01247-3 29057953 PMC5651836

[50] Langston J.A., Shaghasi T., Abbate E., Xu F., Vlasenko E. and Sweeney M.D. (2011) Oxidoreductive cellulose depolymerization by the enzymes cellobiose dehydrogenase and glycoside hydrolase 61. Appl. Environ. Microbiol. 77, 7007–7015 10.1128/AEM.05815-11 21821740 PMC3187118

[51] Sygmund C., Kracher D., Scheiblbrandner S., Zahma K., Felice A.K., Harreither W. et al. (2012) Characterization of the two *Neurospora crassa* cellobiose dehydrogenases and their connection to oxidative cellulose degradation. Appl. Environ. Microbiol. 78, 6161–6171 10.1128/AEM.01503-12 22729546 PMC3416632

[52] Garajova S., Mathieu Y., Beccia M.R., Bennati-Granier C., Biaso F., Fanuel M. et al. (2016) Single-domain flavoenzymes trigger lytic polysaccharide monooxygenases for oxidative degradation of cellulose. Sci. Rep. 6, 28276 10.1038/srep28276 27312718 PMC4911613

[53] Varnai A., Umezawa K., Yoshida M. and Eijsink V.G.H. (2018) The pyrroloquinoline-quinone-dependent pyranose dehydrogenase from *Coprinopsis cinerea* drives lytic polysaccharide monooxygenase action. Appl. Environ. Microbiol. 84, e00156–18 10.1128/AEM.00156-18 29602785 PMC5960967

[54] Cannella D., Mollers K.B., Frigaard N.U., Jensen P.E., Bjerrum M.J., Johansen K.S. et al. (2016) Light-driven oxidation of polysaccharides by photosynthetic pigments and a metalloenzyme. Nat. Commun. 7, 11134 10.1038/ncomms11134 27041218 PMC4822002

[55] Bissaro B., Kommedal E., Rohr A.K. and Eijsink V.G.H. (2020) Controlled depolymerization of cellulose by light-driven lytic polysaccharide oxygenases. Nat. Commun. 11, 890 10.1038/s41467-020-14744-9 32060276 PMC7021734

[56] Bissaro B., Forsberg Z., Ni Y., Hollmann F., Vaaje-Kolstad G. and Eijsink V.G.H. (2016) Fueling biomass-degrading oxidative enzymes by light-driven water oxidation. Green Chem. 18, 5357–5366 10.1039/C6GC01666A

[57] Kommedal E.G., Saether F., Hahn T. and Eijsink V.G.H. (2022) Natural photoredox catalysts promote light-driven lytic polysaccharide monooxygenase reactions and enzymatic turnover of biomass. Proc. Natl. Acad. Sci. U.S.A. 119, e2204510119 10.1073/pnas.2204510119 35969781 PMC9407654

[58] Stepnov A.A., Christensen I.A., Forsberg Z., Aachmann F.L., Courtade G. and Eijsink V.G.H. (2022) The impact of reductants on the catalytic efficiency of a lytic polysaccharide monooxygenase and the special role of dehydroascorbic acid. FEBS Lett. 596, 53–70 10.1002/1873-3468.14246 34845720

[59] Dimarogona M., Topakas E., Olsson L. and Christakopoulos P. (2012) Lignin boosts the cellulase performance of a GH-61 enzyme from *Sporotrichum thermophile*. Bioresour. Technol. 110, 480–487 10.1016/j.biortech.2012.01.116 22342036

[60] Bennati-Granier C., Garajova S., Champion C., Grisel S., Haon M., Zhou S. et al. (2015) Substrate specificity and regioselectivity of fungal AA9 lytic polysaccharide monooxygenases secreted by *Podospora anserina*. Biotechnol. Biofuels 8, 90 10.1186/s13068-015-0274-3 26136828 PMC4487207

[61] Mollers K.B., Mikkelsen H., Simonsen T.I., Cannella D., Johansen K.S., Bjerrum M.J. et al. (2017) On the formation and role of reactive oxygen species in light-driven LPMO oxidation of phosphoric acid swollen cellulose. Carbohydr. Res. 448, 182–186 10.1016/j.carres.2017.03.013 28335986

[62] Kracher D. and Ludwig R. (2016) Cellobiose dehydrogenase: an essential enzyme for lignocellulose degradation in nature – a review / Cellobiosedehydrogenase: ein essentielles enzym für den lignozelluloseabbau in der natur – eine übersicht. Die Bodenkultur: J. Land Management, Food and Environment 67, 145–163 10.1515/boku-2016-0013

[63] Henriksson G., Johansson G. and Pettersson G. (2000) A critical review of cellobiose dehydrogenases. J. Biotechnol. 78, 93–113 10.1016/S0168-1656(00)00206-6 10725534

[64] Tan T.C., Kracher D., Gandini R., Sygmund C., Kittl R., Haltrich D. et al. (2015) Structural basis for cellobiose dehydrogenase action during oxidative cellulose degradation. Nat. Commun. 6, 7542 10.1038/ncomms8542 26151670 PMC4507011

[65] Zamocky M., Ludwig R., Peterbauer C., Hallberg B.M., Divne C., Nicholls P. et al. (2006) Cellobiose dehydrogenase–a flavocytochrome from wood-degrading, phytopathogenic and saprotropic fungi. Curr. Protein Pept. Sci. 7, 255–280 10.2174/138920306777452367 16787264

[66] Kremer S.M. and Wood P.M. (1992) Cellobiose oxidase from *Phanerochaete chrysosporium* as a source of Fenton's reagent. Biochem. Soc. Trans. 20, 110S 10.1042/bst020110s 1327893

[67] Mason M.G., Nicholls P. and Wilson M.T. (2003) Rotting by radicals–the role of cellobiose oxidoreductase? Biochem. Soc. Trans. 31, 1335–1336 10.1042/bst0311335 14641057

[68] Li X., Beeson W.T.t., Phillips C.M., Marletta M.A. and Cate J.H. (2012) Structural basis for substrate targeting and catalysis by fungal polysaccharide monooxygenases. Structure 20, 1051–1061 10.1016/j.str.2012.04.002 22578542 PMC3753108

[69] Kracher D., Zahma K., Schulz C., Sygmund C., Gorton L. and Ludwig R. (2015) Inter-domain electron transfer in cellobiose dehydrogenase: modulation by pH and divalent cations. FEBS J. 282, 3136–3148 10.1111/febs.13310 25913436 PMC4676925

[70] Courtade G., Wimmer R., Rohr A.K., Preims M., Felice A.K., Dimarogona M. et al. (2016) Interactions of a fungal lytic polysaccharide monooxygenase with beta-glucan substrates and cellobiose dehydrogenase. Proc. Natl. Acad. Sci. U.S.A. 113, 5922–5927 10.1073/pnas.1602566113 27152023 PMC4889390

[71] Laurent C., Breslmayr E., Tunega D., Ludwig R. and Oostenbrink C. (2019) Interaction between cellobiose dehydrogenase and lytic polysaccharide monooxygenase. Biochemistry 58, 1226–1235 10.1021/acs.biochem.8b01178 30715860 PMC6404106

[72] Hedison T.M., Breslmayr E., Shanmugam M., Karnpakdee K., Heyes D.J., Green A.P. et al. (2021) Insights into the H_2_O_2_-driven catalytic mechanism of fungal lytic polysaccharide monooxygenases. FEBS J. 288, 4115–4128 10.1111/febs.15704 33411405 PMC8359147

[73] Paradisi A., Johnston E.M., Tovborg M., Nicoll C.R., Ciano L., Dowle A. et al. (2019) Formation of a copper(II)–tyrosyl complex at the active site of lytic polysaccharide monooxygenases following oxidation by H_2_O_2_. J. Am. Chem. Soc. 141, 18585–18599 10.1021/jacs.9b09833 31675221 PMC7007232

[74] Jones S.M., Transue W.J., Meier K.K., Kelemen B. and Solomon E.I. (2020) Kinetic analysis of amino acid radicals formed in H_2_O_2_-driven Cu(I) LPMO reoxidation implicates dominant homolytic reactivity. Proc. Natl. Acad. Sci. U.S.A. 117, 11916–11922 10.1073/pnas.1922499117 32414932 PMC7275769

[75] Matsumura H., Umezawa K., Takeda K., Sugimoto N., Ishida T., Samejima M. et al. (2014) Discovery of a eukaryotic pyrroloquinoline quinone-dependent oxidoreductase belonging to a new auxiliary activity family in the database of carbohydrate-active enzymes. PloS ONE 9, e104851 10.1371/journal.pone.0104851 25121592 PMC4133262

[76] Mukai K., Ouchi A., Nagaoka S.-i., Nakano M. and Ikemoto K. (2016) Pyrroloquinoline quinone (PQQ) is reduced to pyrroloquinoline quinol (PQQH_2_) by vitamin C, and PQQH_2_ produced is recycled to PQQ by air oxidation in buffer solution at pH 7.4. Biosci. Biotechnol. Biochem. 80, 178–187 10.1080/09168451.2015.1072462 26264520

[77] Haddad Momeni M., Fredslund F., Bissaro B., Raji O., Vuong T.V., Meier S. et al. (2021) Discovery of fungal oligosaccharide-oxidising flavo-enzymes with previously unknown substrates, redox-activity profiles and interplay with LPMOs. Nat. Commun. 12, 2132 10.1038/s41467-021-22372-0 33837197 PMC8035211

[78] Gardner J.G., Crouch L., Labourel A., Forsberg Z., Bukhman Y.V., Vaaje-Kolstad G. et al. (2014) Systems biology defines the biological significance of redox-active proteins during cellulose degradation in an aerobic bacterium. Mol. Microbiol. 94, 1121–1133 10.1111/mmi.12821 25294408

[79] Tuveng T.R., Arntzen M.O., Bengtsson O., Gardner J.G., Vaaje-Kolstad G. and Eijsink V.G. (2016) Proteomic investigation of the secretome of *Cellvibrio japonicus* during growth on chitin. Proteomics 16, 1904–1914 10.1002/pmic.201500419 27169553

[80] Vincent F., Molin D.D., Weiner R.M., Bourne Y. and Henrissat B. (2010) Structure of a polyisoprenoid binding domain from *Saccharophagus degradans* implicated in plant cell wall breakdown. FEBS Lett. 584, 1577–1584 10.1016/j.febslet.2010.03.015 20227408

[81] Branch J., Rajagopal B.S., Paradisi A., Yates N., Lindley P.J., Smith J. et al. (2021) *C*-type cytochrome-initiated reduction of bacterial lytic polysaccharide monooxygenases. Biochem. J. 478, 2927–2944 10.1042/BCJ20210376 34240737 PMC8981238

